# Targeting the Ubiquitin–Proteasome System in Atrial Fibrillation: Mechanistic Insights and Translational Perspectives

**DOI:** 10.3390/cimb48010046

**Published:** 2025-12-29

**Authors:** Runze Huang, Zhipeng Pu, Zhangrong Chen

**Affiliations:** 1 Department of Cardiovascular Medicine, The Affiliated Hospital of Guizhou Medical University, Guiyang 550004, China; 2 The Key Laboratory of Myocardial Remodeling Research, The Affiliated Hospital of Guizhou Medical University, Guiyang 550004, China; 18722931255@163.com

**Keywords:** ubiquitin–proteasome system, atrial fibrillation, therapeutic target, biomarker, cardiovascular disease

## Abstract

Atrial fibrillation (AF) is the most common sustained arrhythmia, and its initiation and progression involve multiple mechanisms, including electrical remodeling, structural remodeling, inflammatory responses, and oxidative stress. In recent years, the ubiquitin–proteasome system (UPS), a central pathway for maintaining intracellular protein homeostasis, has attracted increasing attention in the pathogenesis of AF. By regulating the degradation and expression of ion channel proteins, Ca^2+^-handling molecules, and pro-fibrotic signaling factors, the UPS plays a pivotal role in key pathological processes such as electrical and structural remodeling. Several E3 ubiquitin ligases (e.g., NEDD4-1/2, MuRF1, WWP1/2, TRAF6), deubiquitinating enzymes (e.g., JOSD2), and immunoproteasome subunits (e.g., β5i) have been shown to exert critical regulatory effects on atrial electrophysiological disturbances, interstitial remodeling, and inflammation. This review provides a comprehensive summary of the regulatory mechanisms of the UPS in AF-associated pathological processes, outlines potential therapeutic targets, and highlights current intervention strategies, including proteasome inhibitors, selective E3 ligase modulators, and natural compounds. Moreover, we discuss the latest advances and future perspectives regarding the application of UPS-based interventions in AF, aiming to provide theoretical foundations and research insights for the mechanistic exploration and innovative therapeutic development of AF.

## 1. Introduction

AF is one of the most common sustained arrhythmias in clinical practice [1], with its incidence increasing significantly with age and closely associated with multiple cardiovascular diseases such as hypertension, coronary artery disease, and heart failure [2]. Data from the Framingham Heart Study (FHS) indicate that the prevalence of AF has tripled over the past 50 years [3,4,5]. However, the high recurrence rate and multifactorial etiology of AF continue to limit the overall effectiveness of current therapeutic strategies. Therefore, elucidating the underlying molecular mechanisms and identifying stable and druggable signaling pathways have become central objectives in AF research and treatment.

Accumulating evidence suggests that AF is a multifactorial and multistep process involving electrical remodeling, structural remodeling, inflammatory responses, and oxidative stress imbalance [6,7,8]. On this basis, intracellular protein homeostasis regulation has gained increasing attention. Protein homeostasis not only governs the synthesis and degradation of ion channels and contractile proteins but also directly modulates cellular stress responses and signal transduction. Among the regulatory pathways, the UPS represents a major selective protein degradation mechanism, playing a key role in stress responses, myocardial injury, remodeling, and repair [9]. Through a cascade of E1, E2, and E3 enzymes, the UPS tags substrate proteins with ubiquitin and directs them to the 26S proteasome for degradation [10,11,12]. This process not only ensures the rapid clearance of misfolded or damaged proteins but also maintains cardiac electrophysiological and structural integrity by regulating the stability of essential functional proteins [13].

In the context of AF, the multifaceted regulatory roles of the UPS have been widely investigated. On the one hand, the UPS contributes to atrial electrical remodeling through the ubiquitination and degradation of potassium channels (e.g., Kv1.5), sodium channels (Nav1.5), and Ca^2+^-handling proteins (e.g., SERCA2a, RyR2). On the other hand, UPS-associated E3 ligases such as MuRF1, the WWP family, and TRAF6 modulate pathways including TGF-β/Smad, AKT, and JNK, thereby deeply participating in atrial fibrosis and structural remodeling. Moreover, the UPS also influences AF progression by regulating inflammatory mediators such as NF-κB and NLRP3, as well as reactive oxygen species (ROS)-induced oxidative damage, further aggravating the atrial microenvironment.

Recent studies have identified multiple UPS-related molecules closely associated with AF, including NEDD4-1/2, MuRF1, WWP1/2, TRAF6, the deubiquitinating enzyme JOSD2, and the immunoproteasome subunit β5i. These molecules exhibit strong biological activity in maintaining atrial electrophysiological stability, structural remodeling, and cellular stress responses, and are thus considered promising therapeutic targets. Parallel to this, a range of regulatory strategies targeting these molecules—such as small-molecule inhibitors, proteasome inhibitors, and natural compounds—have been explored in preclinical and clinical studies, laying the groundwork for linking mechanistic insights to therapeutic interventions.

Therefore, this review focuses on the role of the UPS in AF pathogenesis. Beginning with its composition and function in the heart, we systematically summarize the molecular mechanisms by which the UPS regulates electrical remodeling, structural remodeling, inflammation, and oxidative stress in AF. Special attention is given to the roles of key E3 ligases and deubiquitinating enzymes in signaling networks, along with a summary of current UPS-targeted therapeutic strategies and preclinical advances. By integrating existing evidence, this review aims to elucidate the critical regulatory axes of the UPS in AF and highlight potential therapeutic pathways, thereby providing a theoretical framework and future directions for advancing mechanistic research and optimizing AF intervention strategies.

### Literature Search and Review Approach

The literature included in this review was identified through a comprehensive search of the PubMed, Web of Science, and Scopus databases up to August 2025. Relevant publications were retrieved using combinations of keywords including “atrial fibrillation”, “ubiquitin–proteasome system”, “ubiquitination”, “E3 ubiquitin ligase”, “deubiquitinating enzymes”, and “proteasome”. Original research articles and review papers focusing on the involvement of the UPS in atrial fibrillation and atrial remodeling were considered. Studies unrelated to cardiovascular disease or not directly relevant to AF pathophysiology were excluded. Additional relevant articles were identified by manual screening of reference lists. This review is intended as a narrative, non-systematic overview of current evidence, with a primary focus on preclinical mechanistic studies, complemented by available human atrial tissue and clinical data where applicable. Given the heterogeneity of experimental models and study designs, a formal systematic review or meta-analysis was not performed.

## 2. Composition and Roles of the UPS in the Heart

The UPS represents a major protein quality control mechanism in cardiomyocytes, acting in concert with the autophagy–lysosome system to maintain cellular proteostasis. It is composed of ubiquitin-activating enzymes (E1), ubiquitin-conjugating enzymes (E2), ubiquitin ligases (E3), and the 26S proteasome [14]. In the heart, the UPS not only regulates the stability of ion channels and Ca^2+^-handling proteins but also plays essential roles in stress responses, metabolic regulation, and cellular repair. Under physiological conditions, experimental studies in cardiomyocytes and animal models have shown that the UPS ensures normal electrical activity and contractile function of cardiomyocytes by rapidly eliminating misfolded or damaged proteins. Beyond its intrinsic proteolytic function, the UPS operates in close coordination with the autophagy–lysosome system to maintain protein homeostasis in cardiomyocytes. Increasing evidence indicates that the UPS and the autophagy–lysosome system are functionally interconnected rather than independent degradative pathways. While the UPS primarily mediates the degradation of short-lived, misfolded, or regulatory proteins, ubiquitination also serves as a key signal for selective autophagy, including aggrephagy and mitophagy. In cardiomyocytes, impairment of proteasomal activity can activate compensatory autophagic responses, whereas dysregulated autophagy may increase the proteostatic burden on the UPS. Disruption of this coordinated balance has been implicated in cardiac stress responses and pathological remodeling.

Within the cardiovascular system, E3 ligases such as the NEDD4 family, the TRIM family, and MuRF1 are of particular importance, as they modulate the onset and progression of cardiovascular diseases through multiple mechanisms. For example, NEDD4-1 mediates the ubiquitination and subsequent degradation of L-type calcium channels, thereby regulating their membrane abundance and function, which in turn governs cardiac excitability [15]. NEDD4-2 regulates atrial electrical activity by controlling the ubiquitination and degradation of potassium channel proteins such as Kv1.5 [16]. Meanwhile, MuRF1 contributes to myocardial remodeling by promoting the degradation of myofibrillar proteins [17].

In response to inflammatory stimuli (e.g., IFN-γ induction), the standard proteasome subunits (β1, β2, β5) can be replaced by immunoproteasome subunits (β1i, β2i, β5i) to form the 20S immunoproteasome, a process with critical implications in cardiovascular disease [18,19]. Notably, the β5i subunit has been shown to promote cardiac hypertrophy, heart failure, and AF by targeting the autophagy-related protein ATG5 for degradation, thereby suppressing autophagic activity. Conversely, inhibition of β5i alleviates angiotensin II–induced cardiac hypertrophy, fibrosis, and inflammatory responses [20,21].

**Figure 1:**
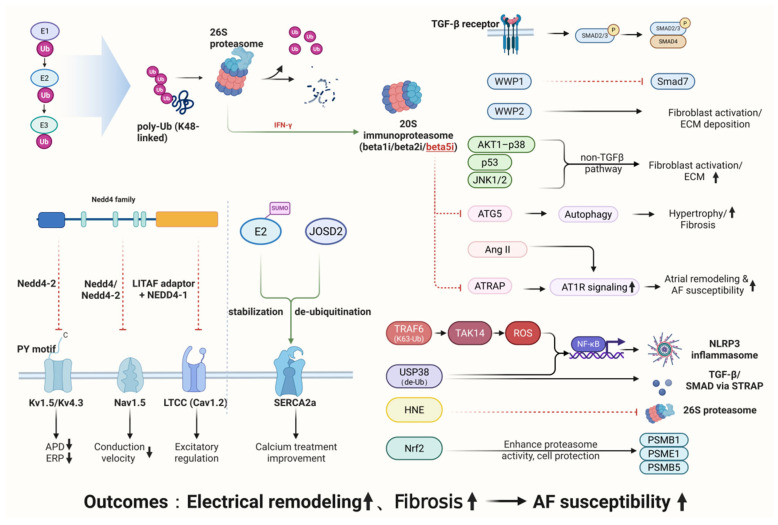
Mechanistic overview of the UPS in AF. The UPS regulates cardiac protein homeostasis through coordinated actions of ubiquitin-activating, -conjugating, and -ligating enzymes, directing substrates to the proteasome for degradation. In AF, dysregulated UPS activity contributes to electrical remodeling, structural remodeling/fibrosis, and inflammation oxidative stress through interconnected pathways involving ion-channel turnover, profibrotic signaling, and immune–inflammatory responses. Together, these processes form an integrated network that promotes atrial remodeling and increases susceptibility to atrial fibrillation. Detailed molecular mechanisms are described in the main text.

Dysregulation of the UPS may lead to aberrant degradation or accumulation of key proteins, thereby driving atrial remodeling. A comprehensive understanding of the specific roles of the UPS in AF pathogenesis is expected to provide a theoretical basis for developing novel therapeutic strategies. Taken together, the UPS serves as a pivotal hub linking protein homeostasis to the pathogenesis of AF. By integrating its roles in ion channel regulation, structural remodeling, inflammation, and oxidative stress, the UPS connects upstream molecular triggers with downstream pathological remodeling (Figure 1 and Table 1). This central position highlights UPS not only as a key mechanistic driver but also as a promising therapeutic target in AF.

## 3. Molecular Links Between UPS Dysregulation and AF Pathogenesis

### 3.1. Mechanisms of UPS in Atrial Electrical Remodeling

Electrical remodeling in AF refers to electrophysiological alterations induced by sustained fibrillatory activity, primarily characterized by shortened action potential duration, reduced effective refractory period, and slowed conduction velocity [22]. These changes facilitate the persistence of AF. Recent in vivo and in vitro studies indicate that UPS dysregulation may play an important role in this process by regulating the stability of various ion channel proteins. It should be noted that the strength of evidence supporting these pathways varies substantially, with most data derived from preclinical models and limited validation in human atrial tissue.

#### 3.1.1. Ubiquitination and Degradation of Potassium Channel Proteins

Potassium channels are central to the repolarization of cardiomyocytes, and their dysfunction leads to altered action potential duration [23]. Studies have shown that potassium channels interact with NEDD4-2 via a conserved C-terminal proline–tyrosine (PY) motif, leading to proteasomal ubiquitination and degradation [24,25,26]. In ex vivo atrial tissues from AF patients, the expression of multiple potassium channel subtypes (e.g., Kv1.5, Kv4.3) is markedly reduced, closely associated with NEDD4-2 activation. Experimental evidence shows that NEDD4-2 C2 knockout (KO) mice exhibit bradycardia at rest, prolonged QRS and QT intervals, shortened PR intervals, and altered heart rate variability. Loss of the NEDD4-2 C2 isoform may impair ubiquitination and degradation of cardiac ion channels, reducing their membrane expression and function, thereby contributing to electrophysiological abnormalities [16]. Inhibition of NEDD4-2 has been suggested to increase Kv1.5 protein levels, prolong action potential duration, and reduce both the inducibility and maintenance of AF.

#### 3.1.2. Regulation of Sodium Channel Proteins

The cardiac sodium channel Nav1.5 is essential for cardiomyocyte excitability and conduction velocity [27,28,29]. Experimental studies suggest that UPS hyperactivation is associated with reduced Nav1.5 expression, which may contribute to atrial electrical remodeling under atrial fibrillation–related conditions. The ubiquitin ligase Nedd4-2 binds to the PY motif of Nav1.5, promoting its ubiquitination and degradation [30]. Beyond NEDD4-2, NEDD4L, another HECT-type E3 ligase, has also been implicated in the ubiquitination of Nav1.5, catalyzing ubiquitin conjugation to intracellular substrates [31,32,33].

#### 3.1.3. UPS-Dependent Dysregulation of Calcium-Handling Proteins

Disturbances in Ca^2+^ handling proteins represent another hallmark of electrical remodeling in AF [34,35]. Ryanodine receptor 2 (RyR2) and sarco/endoplasmic reticulum Ca^2+^ ATPase (SERCA2a) are two key regulators of Ca^2+^ cycling in cardiomyocytes [36,37]. UPS has been shown to modulate the stability of these Ca^2+^-handling proteins in animal models and cultured cardiomyocytes, thereby influencing AF progression [38]. SUMOylation has emerged as an additional regulatory mechanism of SERCA2a, enhancing its Ca^2+^ uptake activity and reducing degradation. In a diet-induced rat obesity model, animals developed cardiac dysfunction accompanied by reduced SERCA2a protein levels and diminished SUMOylation, likely due to downregulation of the conjugating enzyme E2 (ubiquitin-conjugating enzyme 9). Furthermore, lipopolysaccharide-induced tumor necrosis factor ligand (LITAF) acts as an adaptor protein to promote NEDD4-1-mediated ubiquitination and subsequent degradation of L-type calcium channels, thereby regulating their membrane abundance and controlling cardiac excitability [15,39].

Accumulating evidence from preclinical models indicates that UPS dysregulation may contribute to electrical remodeling by regulating the stability of multiple ion channel proteins. These maladaptive changes establish a substrate that not only perpetuates electrical instability but also interacts with downstream pathways such as structural remodeling and fibrotic signaling. Thus, UPS dysfunction serves as a mechanistic bridge linking electrical remodeling to broader pathological remodeling processes in AF, paving the way for subsequent structural, inflammatory, and oxidative alterations.

### 3.2. Mechanisms of UPS in Structural Remodeling of Atrial Fibrillation

Structural remodeling in AF is characterized by alterations in tissue architecture and cellular ultrastructure, leading to atrial dilation and fibrosis [40]. Atrial fibrosis plays a central role in this process, resulting from excessive deposition of extracellular matrix (ECM) proteins within the myocardial interstitium. These structural changes predispose patients to conduction defects that facilitate reentry and rotor formation, thereby sustaining AF [41]. The UPS exerts dual and context-dependent effects in this process—by regulating the stability of multiple structural proteins and signaling molecules, it can either promote pathological remodeling or activate compensatory protective mechanisms. These conclusions are primarily derived from in vivo animal models, supported by in vitro mechanistic studies in cardiomyocytes and cardiac fibroblasts.

#### 3.2.1. Roles of the UPS in AF

The UPS influences cardiac fibrosis primarily through the transforming growth factor-β (TGF-β) signaling pathway, targeting key components such as TGF-β receptors, the Smad2/3/4 complex, and the inhibitory Smad7. In addition, it modulates TGF-β–independent profibrotic pathways, including p53, AKT1-p38, and JNK1/2 cascades [42,43]. Notably, the effects of the UPS in cardiac fibrosis are not uniform. Certain enzymes exhibit either profibrotic or antifibrotic activities depending on the cellular context. For example, WWP and MuRF1 display dual and sometimes opposing functions in myocardial fibrosis. The reported roles of MuRF1 appear to be highly context-dependent. While several studies associate MuRF1 upregulation with pathological remodeling and contractile dysfunction, other evidence suggests that MuRF1 may exert adaptive or protective effects under specific metabolic or stress conditions. These discrepancies likely reflect differences in experimental models, disease stage, and tissue specificity, as most available data are derived from ventricular or non-AF models rather than human atrial tissue. This functional diversity may arise from variations in substrate specificity among different E3 ligases, the distinct molecular pathways involved in various types of cardiac fibrosis, or differences in subcellular localization and molecular interactions of these enzymes within specific cardiac cell types.

(1) TGF-β/Smad-Dependent Pathway

The UPS regulates cardiac fibrosis by ubiquitinating TGF-β receptors and their downstream Smad2/3/4 complex, while promoting the degradation of the inhibitory molecule Smad7, thereby enhancing fibroblast activation and collagen deposition. E3 ubiquitin ligases WWP1 and WWP2 have been shown to augment TGF-β signaling—WWP1 indirectly upregulates the TGF-β/Smad pathway via Smad7 degradation [44,45], whereas WWP2 directly promotes fibroblast activation, suggesting their potential as therapeutic targets for antifibrotic intervention [46,47]. Nevertheless, the functional roles of WWP1 and WWP2 show notable heterogeneity across studies. Their reported profibrotic effects appear to be strongly influenced by cell type, experimental context, and disease model, and direct evidence from human atrial tissue remains limited. Therefore, their precise contributions to atrial fibrosis in AF should be interpreted with caution.

(2) Non-TGF-β Pathways

The proteasome system also modulates fibrosis through TGF-β-independent signaling cascades, including p53, AKT1-p38, and JNK1/2, which regulate fibroblast proliferation and ECM accumulation. For example, MuRF1 maintains myocardial homeostasis during compensatory hypertrophy by degrading myofibrillar proteins; however, its overexpression has been linked to atrial dilation and contractile dysfunction in AF patients, indicating its dual role in cardiac remodeling [17].

(3) Stress and Inflammatory Regulation

TRAF6 promotes cardiac remodeling via ROS-dependent self-ubiquitination that activates TAK1, amplifying oxidative stress and inflammatory responses [48,49]. Conversely, the E3 ligase TRIM16 mitigates hypertrophic remodeling by suppressing oxidative stress through downregulation of the Prdx1/Nrf2 pathway [50].

(4) Emerging Regulatory Factors

Recent studies have identified several novel UPS-associated modulators of cardiac remodeling. FBXW7 promotes myocardial hypertrophy through the EZH2-SIX1 signaling axis [51], whereas overexpression of RNF5 alleviates NF-κB-mediated inflammation and suppresses fibrotic progression [52].

#### 3.2.2. Deubiquitinases in the Regulation of Atrial Fibrillation

DUBs play a crucial role in maintaining substrate protein stability and are increasingly recognized as key regulators in the pathophysiology of AF. Recent studies have shown that JOSD2 mediates the deubiquitination of SERCA2a, thereby enhancing its stability. Through modulation of SERCA2a, JOSD2 deficiency disrupts calcium handling and promotes hypertrophy in primary cardiomyocytes, contributing to the initiation and progression of AF [53,54]. In addition, USP38 has been identified as a critical mediator of chronic kidney disease (CKD)-associated AF. By stabilizing STRAP and activating the TGF-β/Smad signaling pathway, USP38 facilitates atrial fibrosis and electrical remodeling. Thus, targeting USP38 may represent a promising therapeutic strategy for CKD-related AF [55,56,57].

#### 3.2.3. Role of the Immunoproteasome in Atrial Fibrillation

Upon stimulation by cytokines such as interferon-γ (IFN-γ), the standard proteasome subunits can be replaced by immunosubunits to form the 20S immunoproteasome. The immunoproteasome exerts diverse biological functions, including the regulation of proinflammatory cytokine production, T cell differentiation and survival, oxidative stress, and muscle mass maintenance. Studies have shown that β5i expression and chymotrypsin-like activity are markedly upregulated in both the atria of angiotensin II (Ang II)–infused mice and in the serum of AF patients. Moreover, β5i modulates Ang II–induced atrial remodeling and AF by decreasing the stability of ATRAP and activating AT1R-mediated signaling. Thus, β5i acts as a key regulatory factor in AF pathogenesis and may represent a promising therapeutic target [21]. It should be noted that immunoproteasome activation, including β5i upregulation, may exert both detrimental and context-dependent adaptive effects, particularly under inflammatory stress. Whether β5i activation represents a maladaptive driver of atrial remodeling or a compensatory response to sustained inflammation remains to be clarified in AF-specific and human atrial studies.

In addition, β5i serves as a critical modulator of autophagy and cardiac hypertrophy by promoting the degradation of ATG5, thereby suppressing autophagic activation and leading to cardiac hypertrophy and dysfunction. This finding highlights an essential compensatory relationship between immunoproteasome activity and autophagy in the development of cardiac hypertrophy [20,58].

#### 3.2.4. UPS-Mediated Inflammatory and Oxidative-Stress Pathways in Atrial Fibrillation

The UPS contributes critically to atrial inflammatory remodeling by regulating key signaling molecules such as nuclear factor-κB (NF-κB) and the NOD-like receptor protein 3 (NLRP3) inflammasome [59,60,61]. Experimental studies have shown that mice with cardiac-specific overexpression of ubiquitin-specific protease 38 (USP38) exhibit pronounced atrial enlargement, aggravated fibrosis, and heightened AF susceptibility under pressure overload. Mechanistically, USP38 directly interacts with and deubiquitinates NF-κB, thereby increasing phosphorylated NF-κB (p-NF-κB) levels, accompanied by upregulation of NLRP3 and downstream inflammatory mediators such as TNF-α, IL-1β, and IL-6 [57]. Activation of the NF-κB/NLRP3 axis by USP38 accelerates adverse atrial remodeling under hemodynamic stress, suggesting USP38 as a potential therapeutic target for pressure-overload-induced AF.

Oxidative stress represents another pivotal pathological driver of AF [62]. Elevated ROS impairs ER and mitochondrial function, leading to the accumulation of oxidized and misfolded proteins that perturb UPS activity [63]. Lipid-peroxidation products such as 4-hydroxy-2-nonenal (HNE) can exacerbate this process by impairing 26S proteasome-dependent proteolysis, thereby increasing intracellular accumulation of oxidatively modified proteins. Conversely, antioxidant transcription factors such as nuclear factor erythroid 2-related factor 2 (Nrf2) enhance proteasome capacity by upregulating PSMB1 and PA28α/PSME1 expression and improving Nrf2 binding to the PSMB5 promoter, which restores proteasomal activity and promotes cell survival [64]. Thus, oxidative stress promotes the accumulation of ubiquitinated proteins by simultaneously increasing ubiquitin-related enzyme expression and suppressing proteasomal degradation, ultimately contributing to myocardial injury.

Although inflammation and oxidative stress act through distinct molecular mechanisms, they are tightly interconnected via the NF-κB signaling hub [65]. ROS-activated NF-κB enhances pro-inflammatory cytokine expression, while inflammatory mediators such as TGF-β1 further stimulate ROS generation, establishing a self-perpetuating feedback loop [66]. This vicious cycle drives both atrial structural and electrical remodeling, forming the pathological substrate for AF. Collectively, evidence from animal models and cellular systems supports a critical role of UPS-mediated inflammatory and oxidative-stress pathways in AF remodeling.

**Table 1:** Key UPS-Regulated Proteins and Their Functions in Atrial Fibrillation.

Regulatory Component	Target Protein/Pathway	Functional Effect (↑ Activation/↓ Inhibition)	Associated Pathological Mechanism
NEDD4-2	Kv1.5/Nav1.5/L-type calcium channel	Shortened action potential duration (↑)/Reduced conduction velocity (↑)	Electrical remodeling
MuRF1	Myofibrillar proteins	Enhanced atrial structural remodeling (↑)	Structural remodeling
WWP1/2	TGFβ/Smad	Activation of fibroblast-mediated fibrosis (↑)	Structural remodeling–Fibrosis
β5i (Immunoproteasome subunit)	ATRAP/ATG5	Fibrosis and inflammation amplification (↑)	Structural remodeling–Inflammatory stress
USP38 (DUB)	STRAP/TGFβ	Fibrosis and electrical uncoupling (↑)	Structural + Electrical remodeling
JOSD2 (DUB)	SERCA2a	Improved calcium handling (↓ Hypertrophy)	Structural remodeling–Hypertrophy (putative)

Note. The meanings of arrows (↑, ↓) are consistent with those defined in Table 2.

## 4. Targeting the UPS for Therapeutic Intervention in Atrial Fibrillation

**Figure 2:**
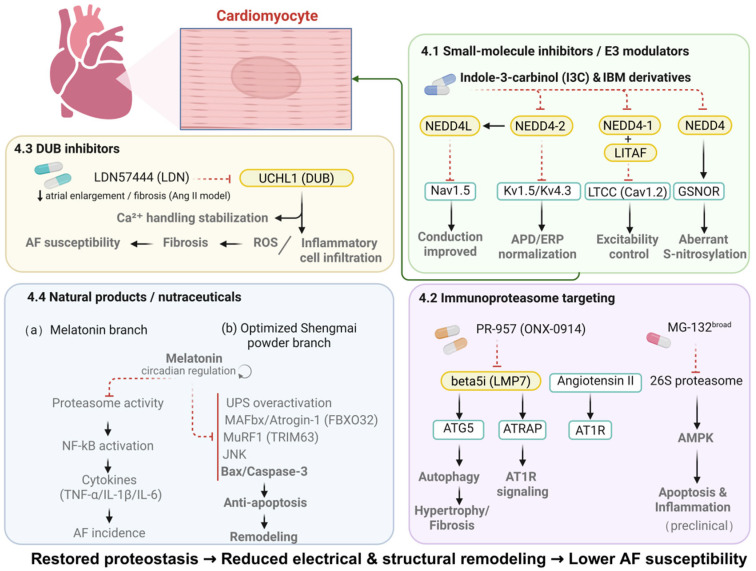
Therapeutic strategies targeting the UPS for AF. The schematic summarizes representative UPS-modulating approaches identified in experimental studies, including small-molecule modulators of E3 ligases, immunoproteasome-targeting strategies, deubiquitinase inhibitors, and selected natural products. These interventions have been shown to influence electrical remodeling, structural remodeling/fibrosis, and inflammatory–oxidative stress pathways in preclinical models. While these strategies provide valuable mechanistic and proof-of-concept insights, their efficacy, safety, and translational relevance in human atrial fibrillation remain to be established. Solid arrows indicate activation, whereas dashed arrows indicate inhibition; ↑ and ↓ denote increased or decreased effects, respectively.

Given the important role of the UPS in AF pathogenesis, multiple UPS-targeted strategies have been explored to interrogate disease mechanisms and to evaluate their potential therapeutic relevance in experimental settings (Figure 2 and Table 2). However, the following discussion focuses primarily on mechanistic insights and experimental feasibility rather than established therapeutic efficacy in atrial fibrillation.

### 4.1. Small-Molecule Inhibitors and E3 Ligase Modulators: Restoring Ion Channel Stability and Electrical Remodeling

The E3 ubiquitin ligase NEDD4 is closely associated with the degradation of Kv1.5, suggesting that its modulation could alleviate electrical remodeling in AF. Targeting specific E3 ligases offers higher molecular specificity and precision. Studies have shown that indole butyric acid methyl ester (IBM) [67,68] and indole-3-carbinol (I3C) [69,70] effectively inhibit NEDD4 activity. In cardiac hypertrophy models, these inhibitors suppress GSNOR ubiquitination and cardiac hypertrophy, maintain GSNOR protein levels, reduce abnormal protein S-nitrosylation, and improve cardiac function. Clinically, indole-3-carbinol has demonstrated promising efficacy as an antitumor agent in phase II clinical trials [67]. These findings suggest that inhibition of NEDD4-mediated ion channel degradation may represent a promising mechanistic strategy; however, its therapeutic relevance for AF remains to be established, as current evidence is largely derived from preclinical and non-AF disease models. However, most existing studies remain at the preclinical or animal-model level, with limited validation in AF-specific models, and further evaluation of safety and off-target effects is warranted.

### 4.2. Immunoproteasome-Specific Targeting: Attenuating the Inflammation–Fibrosis Axis

β5i has been identified as a critical regulatory subunit in AF, and its inhibition holds promise for alleviating cardiac fibrosis and inflammation. The proteasome inhibitor MG-132 has been shown to modulate apoptosis and inflammation through the AMPK signaling pathway in murine models of myocarditis, thereby improving hemodynamic function and suppressing ventricular structural remodeling [71,72]. Selective inhibitors of the immunoproteasome subunit β5i, such as PR-957, have demonstrated favorable effects in reducing cardiac fibrosis and inflammation in experimental models; however, these effects have not yet been validated in human atrial tissue or in clinical AF studies. These agents selectively suppress aberrant proteasome activity under inflammatory conditions, attenuate oxidative stress, and mitigate atrial remodeling [73]. However, these compounds are still in preclinical stages, and further studies are required to clarify their safety, specificity, and translational potential in AF treatment.

### 4.3. Deubiquitinase Inhibitors: Modulating Calcium Handling and AF Susceptibility

Deubiquitinases (DUBs) contribute to AF pathogenesis through multiple mechanisms, including dysregulated calcium handling and increased arrhythmogenic susceptibility. Targeting DUBs thus represents a novel therapeutic avenue for AF. In male wild-type mice, administration of the UCHL1 inhibitor LDN57444 (40 μg/kg) combined with continuous angiotensin II infusion (2000 ng/kg/min) for three weeks markedly reduced left atrial dilation, fibrosis, inflammatory cell infiltration, and reactive oxygen species (ROS) generation. These findings suggest that LDN57444 may serve as a potential therapeutic approach for hypertension-associated AF [74]. Nonetheless, such strategies remain at the animal-experiment stage, and further preclinical and clinical validation is necessary.

### 4.4. Natural Compounds and Nutritional Interventions: Supporting UPS Homeostasis and AF Prevention

A variety of natural compounds and nutritional agents have been shown to assist in restoring UPS homeostasis and preventing AF. Melatonin, for instance, effectively suppresses angiotensin II–induced AF by inhibiting proteasome activity [72]. Its cardioprotective effect is likely mediated by suppression of NF-κB activation and reduction in pro-inflammatory cytokine release, providing a new paradigm for UPS-targeted natural therapies [75]. Furthermore, optimized formulations of Shengmai Powder have demonstrated cardioprotective and antiapoptotic properties by suppressing excessive UPS activation, downregulating MAFbx and MuRF1 overexpression, inhibiting JNK pathway activation, increasing Bcl-2 expression, and reducing Bax and caspase-3 levels [76]. These findings indicate that the agent has progressed from mechanistic studies toward clinical application and may emerge as an effective adjunctive strategy for AF treatment in the near future.

**Table 2:** Current UPS-Related Therapeutic Strategies and Their Research Stages.

Target Category	Representative Agent	Mechanism of Action	Research Stage	Main Effect (↑ Promotes/↓ Inhibits)
E3 ligase inhibitor	IBM/I3C	Inhibits NEDD4-mediated degradation of GSNOR	degradation of GSNOR Phase II clinical trial	↓ Cardiac hypertrophy; improves electrical remodeling
DUB inhibitor	UCHL1 inhibitor LDN57444 (LDN)	Suppresses Ang II–induced fibrosis, inflammation, and ROS production	Animal model study	↓ Atrial fibrosis and inflammation
DUB	USP38	Stabilizes STRAP and activates TGF-β/Smad fibrotic signaling	Animal model (CKD-AF)	↑ Fibrosis and electrical uncoupling
Immunoproteasome inhibitor	PR-957	Selectively inhibits β5i subunit, reducing oxidative stress and fibrosis	Preclinical (AF model)	↓ Atrial remodeling and inflammation
Natural compound	Optimized Shengmai Powder	Suppresses excessive UPS activation; downregulates MAFbx and MuRF1 expression	Basic → Clinical translation	↓ Fibrosis; improves cardiac remodeling

Note: ↑ indicates promotion of cardiac fibrosis or electrical uncoupling; ↓ indicates inhibition of cardiac fibrosis or inflammation. Importantly, none of the UPS-targeted agents listed in Table 2 have been validated in human atrial tissue or clinically tested in patients with atrial fibrillation to date.

### 4.5. Translational Challenges and Limitations of UPS-Targeted Therapies

Despite encouraging mechanistic insights, several major barriers limit the translation of UPS-targeted strategies into AF therapy. First, many UPS components are ubiquitously expressed, raising concerns regarding systemic toxicity and off-target effects. Second, current pharmacological inhibitors often lack atrial or cell-type specificity, which may disrupt essential proteostatic functions in non-cardiac tissues. Third, most available evidence is derived from in vitro systems or non-AF animal models, with limited validation in human atrial tissue. Finally, long-term modulation of the UPS may interfere with adaptive stress responses, underscoring the need for precise, context-dependent therapeutic strategies. Addressing these challenges will be critical for advancing UPS-based interventions toward clinical application in AF. To date, no UPS-targeted therapy has been clinically tested in atrial fibrillation. However, clinical experience from other disease areas, particularly oncology and autoimmune disorders, provides indirect insights into the feasibility and risks of UPS modulation in humans. For example, proteasome inhibitors have demonstrated therapeutic efficacy in hematological malignancies, but their use is frequently associated with systemic toxicity, immunological disturbances, and off-target effects, highlighting potential challenges for cardiovascular applications.

### 4.6. Mechanistic Versus Potentially Translatable UPS Targets in Atrial Fibrillation

Although numerous components of the UPS have been implicated in AF, the strength of supporting evidence and translational relevance differ markedly among targets. Many UPS-related pathways described above, including NEDD4 family ligases, MuRF1, and several deubiquitinating enzymes, have been identified mainly through in vitro experiments or small-animal studies, often in ventricular or non-AF disease models. These findings provide important mechanistic insights but cannot yet be directly extrapolated to human atrial pathophysiology.

In contrast, alterations in the immunoproteasome, particularly the β5i subunit, have been more consistently linked to cardiac inflammation and fibrosis across multiple experimental settings, suggesting relatively higher translational plausibility. Nevertheless, direct validation in human atrial tissue and AF-specific models remains limited, and causal roles in AF have yet to be established.

Taken together, this evidence-weighted stratification suggests that most UPS-related pathways in AF currently remain mechanistic or hypothesis-generating, whereas a limited subset, such as immunoproteasome-related pathways, may warrant further investigation for translational relevance once atrial-specific validation becomes available.

## 5. Future Perspectives

As the core regulatory system of cellular protein homeostasis, the UPS precisely modulates ion channels, fibrotic signaling, inflammation, and oxidative stress, thereby deeply participating in both electrical and structural remodeling in AF. Despite significant mechanistic advances, several critical challenges remain in translating UPS-related findings into clinical applications for atrial fibrillation. Future progress toward bedside application will require atrial-specific validation, improved target selectivity, and rigorous safety evaluation, rather than direct extrapolation from existing preclinical data.

(1) Selectivity and Safety of UPS Targets

Key E3 ligases and deubiquitinases are broadly expressed across multiple tissues; systemic modulation may lead to significant off-target effects. Future work should focus on developing tissue-specific and pathology-dependent strategies for precise UPS regulation.

(2) Lack of AF-Specific Animal Models and Translational Studies

Current evidence is largely derived from murine and rat models whose electrophysiological characteristics differ markedly from those of humans. The absence of large-animal AF models for validating UPS-targeted therapies limits translational feasibility. Establishing such models will be essential for bridging basic research and clinical application.

(3) Complexity of the UPS Regulatory Network and the Challenge of Individualized Therapy

The UPS involves multilayered post-translational modifications and cell-type-specific regulatory patterns. Future research should integrate single-cell omics, spatial transcriptomics, and proteomics to delineate the spatiotemporal regulation of the UPS within the AF microenvironment, identify key nodal regulators, and enable personalized therapeutic interventions.

(4) Future Research Directions

Efforts should concentrate on precision targeting and translational breakthroughs. On one hand, future efforts should prioritize rational target selection, atrial-specific delivery strategies, and integration of multi-omics data. On the other hand, integrating multi-omics data with clinical validation will be crucial for promoting the systematic translation of UPS research from mechanistic insight to therapeutic strategy.

(5) Integration of UPS-Targeted Strategies into Current AF Management

Although current AF management relies primarily on antiarrhythmic drugs, catheter ablation, and upstream therapies targeting comorbidities, these approaches have well-recognized limitations, including incomplete efficacy, procedural recurrence, and limited impact on underlying atrial remodeling. In this context, modulation of the UPS may represent a complementary strategy rather than a standalone therapy.

In contrast to conventional antiarrhythmic drugs, which primarily exert their effects through direct modulation of ion-channel activity and electrophysiological properties, UPS-targeted approaches aim to regulate upstream processes such as protein turnover, inflammatory signaling, and fibrotic remodeling. While antiarrhythmic drugs can be effective for rhythm control, their clinical utility is often constrained by proarrhythmic risk, systemic side effects, and reduced efficacy in the presence of advanced atrial substrate remodeling.

From this perspective, UPS modulation may offer a conceptually distinct and potentially complementary approach by targeting the molecular mechanisms that underlie atrial substrate progression, rather than acutely altering electrical activity. Nevertheless, whether such theoretical advantages translate into improved clinical outcomes remains to be determined and will require rigorous atrial-specific and phenotype-driven investigation.

By targeting proteostasis, inflammatory signaling, and fibrotic remodeling, UPS-based interventions could theoretically enhance the durability of rhythm control, improve atrial substrate modification, or increase responsiveness to conventional therapies. For example, UPS modulation may help stabilize ion-channel homeostasis in patients with drug-refractory electrical remodeling or attenuate progressive fibrosis that limits the long-term success of catheter ablation.

Importantly, UPS-targeted strategies may be particularly relevant in specific clinical phenotypes characterized by heightened inflammatory or metabolic stress, such as AF associated with hypertension, chronic kidney disease, metabolic disorders, or systemic inflammation. These conditions are often accompanied by exaggerated proteostatic imbalance and atrial remodeling, potentially creating a biological context in which UPS modulation could provide incremental benefit.

Nevertheless, the clinical integration of UPS-based approaches remains speculative at present. Future studies should focus on identifying AF phenotypes most likely to benefit from UPS modulation, evaluating combinatorial strategies with existing therapies, and establishing safety and efficacy in atrial-specific and clinically relevant settings.

## 6. Summary

As a key regulatory hub of cellular protein quality control, UPS plays a pivotal role in the pathogenesis of AF, integrating multiple pathological axes, including electrical remodeling, structural remodeling, inflammation, and oxidative stress. This review synthesizes recent evidence to highlight how coordinated actions of E3 ligases, deubiquitinases, and immunoproteasome components contribute to atrial remodeling at multiple levels. While these insights substantially advance our mechanistic understanding of AF, the translation of UPS-targeted strategies into therapy will require further validation in atrial-specific and clinically relevant models.

## Data Availability

No new data were created or analyzed in this study. Data sharing is not applicable to this article.

## Abbreviations

AF	Atrial fibrillation
UPS	Ubiquitin–proteasome system
TGF-β	Transforming growth factor-β
NF-κB	Nuclear factor-κB
USP38	Ubiquitin-specific protease 38
SERCA2a	Sarcoplasmic/Endoplasmic Reticulum Ca^2+^-ATPase 2a
NEDD4-1	Neural precursor cell expressed, developmentally down-regulated 4-1
NEDD4-2	Neural precursor cell expressed, developmentally down-regulated 4-2
WWP1	WW domain-containing E3 ubiquitin ligase 1
WWP2	WW domain-containing E3 ubiquitin ligase 2
TRAF6	TNF receptor-associated factor 6
JOSD2	Josephin domain–containing protein 2
NLRP3	NOD-like receptor family pyrin domain–containing 3
ROS	Reactive oxygen species
ATRAP	Angiotensin II type-1 receptor–associated protein
STRAP	Serine/threonine kinase receptor-associated protein
ATG5	Autophagy-related protein 5
DUBs	Deubiquitinases
GSNOR	S-nitrosoglutathione reductase
I3C	Indole-3-carbinol
LITAF	LPS-induced TNF factor
UCHL1	Ubiquitin C-terminal hydrolase L1
